# Editorial: Pathogenesis and management of glomerular diseases

**DOI:** 10.3389/fmed.2022.1018776

**Published:** 2022-09-26

**Authors:** Sophia Lionaki, Vimal K. Derebail

**Affiliations:** ^1^Department of Nephrology, National and Kapodistrian University of Athens, Attikon University Hospital, Athens, Greece; ^2^UNC Kidney Center, Division of Nephrology and Hypertension, University of North Carolina at Chapel Hill, Chapel Hill, NC, United States

**Keywords:** glomerular diseases, pathogenesis, management—healthcare, outcome, predictor

Glomerular diseases are considered to be the result of inherited or acquired disorders and may manifest in a variety of clinical syndromes, including numerous pictures in terms of severity. A significant number of patients have no symptoms while others discover urinary abnormalities in routine screenings or they may experience low grade symptoms, such as macroscopic hematuria and edema in the lower extremities. Occasional patients present with rapidly progressive glomerulonephritis, a serious condition which may end up in advanced or end-stage kidney disease if remain untreated. Renal histopathology evaluation in combination with the characteristics of the clinical syndrome remains the cornerstone for accurate diagnosis and evidence-based treatment. During the past decade substantial progress has been made in this field, especially regarding the etiology and pathogenesis of these diseases. Recent knowledge has been added, including molecular mechanisms, genetic associations and immunologically-mediated forms of glomerulonephritis, underlining the autoimmune basis associated with genetic risk factors and environmental stimulus leading to immune-mediated injury of the glomeruli (1, 2). The role of animal models studies has been significant in this part, supporting a translational model of how immune reactions mediate the glomerular lesion, which is identified by renal pathologists in the biopsy specimen (1). It has been postulated and presented by several researchers that immune responses to infective agents and self-antigens are implicated in most forms of glomerulonephritis. Toll-like receptors and complement substances are considered to take part in these immune responses, while monocytes and resident glomerular cells are being activated initiating inflammatory reactions in the glomeruli (1). Several lesions in the glomerular filtration barrier are being developed, allowing passage of red blood cells and protein from the blood space (glomerular capillary lumen) into the urine. Therefore, microscopic hematuria, with or without proteinuria, is one of the most common clinical manifestations of glomerular diseases, including IgA nephropathy (IgAN), ANCA-vasculitis, and lupus nephritis (3–8). Clinicians with long standing experience in the field of glomerular diseases regard persistent hematuria as a sign of continuing disease activity, although there are nephrologists who consider microscopic hematuria as a non-threatening finding, resulting from previous damage (8–10). However, the role of microhematuria in the development of glomerular diseases and progressive kidney damage lately received more consideration, since it was shown to be independently associated with progression of renal disease in patients with IgAN (11–13). Importantly, in this Research Topic of *Frontiers in Medicine*, He, Yu et al. report that a higher level of initial hematuria was a remarkable predictor of relapse in patients with primary membranous nephropathy (MN), while the degree and persistence of microscopic hematuria were independently associated with kidney disease progression. The vast majority of patients with primary MN (80%) present with nephrotic-range proteinuria, the remaining 20% have subnephrotic proteinuria while microhematuria appears in ~50–60% of patients overall (14–19). As shown from this study, the hazards of kidney disease progression in patients with idiopathic MN and persistent hematuria was significantly higher than those with non-persistent hematuria during follow-up. As said above glomerular hematuria is caused by injuries in the glomerular filtration barrier structure. In patients with glomerular inflammation infiltrating inflammatory cells, such as leukocytes may release metalloproteinases and reactive oxygen species and the glomerular basement membrane becomes more susceptible to ruptures (4, 20, 21). On the other hand, persistent glomerular hematuria may stimulate renal damage *via* the oxidative stress caused by the release of hemoglobin and iron which is realized from broken erythrocytes into renal tubular cells (4, 10, 21–24). Yet, remission of hematuria was independently associated with reduced risk for renal progression (37%) compared with patients with ongoing microhematuria. Therefore, hematuria diminution may be a predictor of renal survival for patients with primary MN although its precise relationship with end-stage kidney disease or death and histolopathological findings requires to be elucidated in prospective studies.

The same group (He, Zha et al.) explored the issue of prognosis in patients with primary MN and subnephrotic proteinuria. As shown by previous investigators, although 20% of patients with primary MN present with subnephrotic range proteinuria, 61% of them later develop nephrotic range proteinuria, usually within the first year (14–19, 25). There is also long-term benefit of persistent subnephrotic proteinuria with renal survival of >80–90% at 10 years (26). More importantly, in practice, it is probable that these patients might not be properly observed and monitored, putting them at advanced risk of developing nephrotic range proteinuria and chronic kidney disease in the long term. In this regard, studying 205 cases with biopsy-proven primary MN and subnephrotic proteinuria, who had a minimum of 18 months of follow-up, the authors found that initial proteinuria was an independent predictor for partial remission, complete remission, and nephrotic proteinuria progression. The degree of the initial microhematuria was also associated with an increased risk of development of nephrotic range proteinuria. Hence, among patients with primary MN and subnephrotic proteinuria, although the overall prognosis was favorable, future detection and treatment of proteinuria plays a critical role.

The role of tertiary lymphoid organs (TLOs) in the pathogenesis of MN and its clinical associations with the disease, was explored by Wang Z-f et al. in 442 patents with biopsy proven MN. It was found that TLOs, which were defined as structures of accumulated lymphoid cells, were frequently discovered in the renal tissue in these patients. The TLOs density correlated with the severity of the clinical picture, i.e., the disease was worse with increasing TLOs. Patients who had TLOs were more likely to have anti-phospholipase A2 receptor autoantibodies in their circulation while patients without TLOs had significantly higher probability to achieve complete remission.

Lupus MN, a type of secondary form of this disease, accounting for 10–20% of total cases of lupus nephritis, is generally associated with a better patient and renal survival, compared to proliferative classes of renal involvement. In this issue of the journal (Kapsia et al.) a report of 27 patients with pure lupus MN, underlined the good prognosis, which is associated with type of lupus glomerulopathy. The patients were followed for a median period of 77 months, all had eGFR > 60 ml/min/1.73 m2 and long-term renal survival was very good.

As expected, the issue of prognosis remains crucial in all patients with glomerular disorders and particularly in IgAN because patients may present with various clinical and histopathological pictures, in terms of severity. In this regard, Dong et al. after studying 1,424 patients with IgAN found that arterial-arteriolar sclerosis was associated with the composite outcome of >50% reduction in estimated glomerular filtration rate, end-stage kidney disease or death and was an independent risk factor for the progression of IgAN. The severity of arterial-arteriolar sclerosis was associated with these outcomes and there was shown a trend that it might serve as an independent risk marker for progression of IgAN. Likewise, Wang Y-N et al. showed that the prevalence of hyperhomocysteinemia was high in patients with IgAN and greater than that seen in other forms of glomerular disease. The homocysteine/eGFR ratio was associated with the histopathologic features of IgAN, including the proportion of global glomerulosclerosis, the proportion of ischemia originated glomerular sclerosis, and the severity of tubular atrophy/interstitial fibrosis. Importantly, this ratio was an independent risk factor for chronic kidney disease progression event.

Finally, one study addressed an important consideration in individuals with glomerular disease, that of pregnancy. Women with glomerular disease may be at particular risk for adverse pregnancy outcomes and at risk for subfertility and early menopause (27). Marinaki et al. describe outcomes in 22 women with glomerular disease who experienced a pregnancy. All patients were in complete or partial remission before conception with well-preserved renal function. Disease relapse and preeclampsia occurred in only 6.9 and 6.7% respectively and preterm births occurred in 23%. Overall adverse events were low, highlighting the importance of ensuring disease quiescence prior to conception.

In this Research Topic of *Frontiers in Medicine*, important associations between the clinical picture, histopathology and prognosis have been reported regarding frequent glomerular diseases. Identification of prognostic indicators among clinical, histopathological, or serological characteristics of patients is still critical in this field, in order to make decisions regarding planning of therapy at initial diagnosis and in the long-term. Until basic science finally move therapy of these important renal diseases from the exclusive reliance on glucocorticoids and toxic generalized immunosuppression to a new era of steroid-free, targeted, personalized renal care with agents that are safer, and more effective than the medications which have been the mainstays of therapy for a long time (28).

## Author contributions

All authors listed have made a substantial, direct, and intellectual contribution to the work and approved it for publication.

## Conflict of interest

The authors declare that the research was conducted in the absence of any commercial or financial relationships that could be construed as a potential conflict of interest.

## Publisher's note

All claims expressed in this article are solely those of the authors and do not necessarily represent those of their affiliated organizations, or those of the publisher, the editors and the reviewers. Any product that may be evaluated in this article, or claim that may be made by its manufacturer, is not guaranteed or endorsed by the publisher.
